# Reciprocal processes of sensory perception and social bonding: an integrated social‐sensory framework of social behavior

**DOI:** 10.1111/gbb.12781

**Published:** 2021-12-14

**Authors:** Nora H. Prior, Ehren J. Bentz, Alexander G. Ophir

**Affiliations:** ^1^ Department of Psychology Cornell University Ithaca New York USA

**Keywords:** autism, birdsong, call, dopamine, female song, nonapeptides, pair‐bond maintenance, social behavior network, social decision‐making network, song control system

## Abstract

Organisms filter the complexity of natural stimuli through their individual sensory and perceptual systems. Such perceptual filtering is particularly important for social stimuli. A shared “social umwelt” allows individuals to respond appropriately to the expected diversity of cues and signals during social interactions. In this way, the behavioral and neurobiological mechanisms of sociality and social bonding cannot be disentangled from perceptual mechanisms and sensory processing. While a degree of embeddedness between social and sensory processes is clear, our dominant theoretical frameworks favor treating the social and sensory processes as distinct. An integrated social‐sensory framework has the potential to greatly expand our understanding of the mechanisms underlying individual variation in social bonding and sociality more broadly. Here we leverage what is known about sensory processing and pair bonding in two common study systems with significant species differences in their umwelt (rodent chemosensation and avian acoustic communication). We primarily highlight that (1) communication is essential for pair bond formation and maintenance, (2) the neural circuits underlying perception, communication and social bonding are integrated, and (3) candidate neuromodulatory mechanisms that regulate pair bonding also impact communication and perception. Finally, we propose approaches and frameworks that more fully integrate sensory processing, communication, and social bonding across levels of analysis: behavioral, neurobiological, and genomic. This perspective raises two key questions: (1) how is social bonding shaped by differences in sensory processing?, and (2) to what extent is sensory processing and the saliency of signals shaped by social interactions and emerging relationships?

## Graphical abstract

Defining the brain networks that support social communication and behavior has tremendous value for elucidating brain function and supporting broad translational applications for human mental health and well‐being. The foundations and trajectories of prairie vole and zebra finch neurobiological research are quite different, but together these two well‐studied species offer complementary perspectives on what an integrated social‐sensory communication circuit might look like. We believe that integrating the social behavior/decision making network with brain regions responsible for chemosensory and auditory processing provides a platform for such research. Designing experiments that focus on how social experience modulates perception, sensory processing, and communication promise to be particularly powerful for clarifying how social bonding produces lasting impacts on brain and behavior.

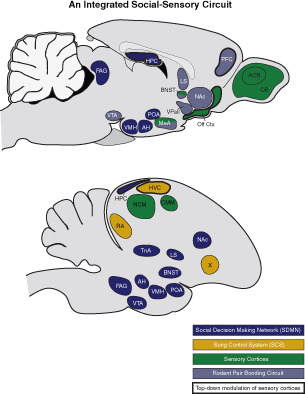

## 1 INTRODUCTION

Our social worlds emerge from, and persist within, our sensory and perceptual experiences. One of the great challenges facing the study of behavior and ethology has been to understand the sensory and perceptual world in which an organism lives. An organism's perceptual space (i.e., the umwelt)^1^ refers to the environmental surroundings to which an animal attends for survival and reproduction. All stimuli, social or otherwise, are filtered by an organism's sensory and perceptual systems. A “social umwelt” allows individuals of any particular species to respond appropriately to the natural diversity of cues and signals they encounter during social interactions with conspecifics. Social interactions occur within, and because of, these shared perceptual spaces. Through repeated social interactions, shared perceptual spaces provide the fundamental basis for the formation and expression of social relationships.^2^, ^3^In this way, the behavioral and neurobiological mechanisms of sociality and social bonding are inextricably linked with perception. However, approaches in both research and clinical settings conceptually, methodologically, and diagnostically delineate behavioral and physiological mechanisms as *either* social or sensory. This modular perspective both reflects and reinforces the significant gaps in our knowledge of how these processes co‐occur and emerge.

Across species, patterns of social behavior reflect and exploit species‐specific differences in perceptual spaces. Many species have exceptional sensory capabilities in certain domains, creating specific 'channels' of communication. For example, many species operate within perceptual spaces that extend beyond the human experience or human perception, such as ultrasonic vocalizations,^4^, ^5^ fine auditory temporal processing,^6^, ^7^ ultra‐violet and infrared color vision,^8^, ^9^, ^10^ and chemosensory perception by the vomeronasal organ.^11^, ^12^ Differences in animal behavior reflect these differences in perceptual abilities, and behavior is often specialized within each modality (e.g., echolocation and hunting in bats).^13^ Key differences across species in social behavior (including parenting, courtship, and mate choice) reflect these sensory differences (e.g., vision for mate choice in primates^14^, ^15^, ^16^ or chemosensation and social recognition in rodents).^17^, ^18^ The co‐evolution of social and sensory systems has been studied in over 70 species, including numerous invertebrates, fish, amphibians, birds, primates, and even whales.^19^, ^20^ The majority of research has focused on co‐evolution of social behavior with visual or auditory systems,^8^, ^19^, ^21^, ^22^ but there is also evidence of co‐evolution for social behaviors with vibratory and chemosensory systems.^19^, ^23^, ^24^, ^25^ Such comparisons across species highlight the potential magnitude of sensory differences, the specializations of key 'primary' modalities (e.g., visual, auditory, or chemosensory), and the embeddedness of social behavior within these sensory systems. Identifying species differences in 'primary' channels of communication is an important first step toward developing a unified framework to understand the reciprocal impacts of sensory and social processes.

### 1.1 An integrated sensory‐social framework of pair bonding

Developing frameworks that reflect the extent to which social and sensory systems are interconnected has the potential to greatly expand our understanding of social bonding. Although it has long been known that communication and perception are essential to social bonding, research at the interface of these processes has been restricted to a few key questions – largely outside of broader pair bonding research. For example, there is extensive research on female mate choice and social recognition. However, there is very little research on how patterns of dynamic communication vary across pairs or how these patterns of communication are shaped over time by pair bonding. Furthermore, even though the original mammalian pair bonding circuit^26^ includes the olfactory bulb, whether differences in sensory processing relate to differences in pair bonding remain unknown. These gaps in our knowledge are not specific to communication. In fact, we know surprisingly little about the mechanisms and consequences underlying individual differences in social bonding across species. We also currently have a poor understanding of how social bonds change over time^27^, ^28^, ^29^, ^30^, ^31^ or how such dynamic relationships produce enduring impacts on individuals' brain and behavior.^32^, ^33^ Such gaps in our understanding have implications for both basic and applied research. The majority of pair bonding research still conceptualizes pair bonding as a dichotomous state (individuals being either bonded or not bonded) rather than considering it as a continuous spectrum of individual differences in bond strength. One approach to deepening our understanding of social bonding broadly is to rely on an integrated social‐sensory framework of bonding. Such a framework would emphasize the more mundane and dynamic aspects of the social interactions that culminate to form and reflect bonds.

In this review, we leverage what is known about sensory processing and pair bonding in two common study systems. We discuss the prairie vole (*Microtus ochrogaster*) and zebra finch (*Taenopygia guttata*) systems to explore such an integrated social‐sensory framework of pair bonding. For each study system, we (1) describe the unique aspects of ethology/ecology for that species, (2) situate this knowledge within the broader research programs in each relevant field, (3) highlight the role of communication and perception in pair bonding, (4) review candidate neural circuits underlying the integration of communication, perception, and pair bonding, and (5) emphasize the extensive potential for neuromodulation of signal processing based on social experience. Prairie voles and zebra finches offer very different insights into how to develop and apply an integrated social‐sensory framework to pair bonding. We conclude by discussing future directions that will extend this integration across behavioral, neurobiological, and genomic levels of analysis.

## 2 PRAIRIE VOLES

*Microtus* voles have become an established system for studying mammalian social structures, reproductive decision‐making, and social behavior. In particular, prairie voles (*M. ochrogaster*) are best known for their impact on understanding the behavioral and neurobiological mechanisms associated with pair bonding and social monogamy.^28^, ^29^, ^34^, ^35^, ^36^ The greater vole study system is particularly powerful because closely related species differ in their levels of social organization, mating systems, and propensity to form social bonds, even though they often experience similar ecological pressures in nature.^37^, ^38^, ^39^, ^40^ Montane and meadow voles (*M. montanus* and *M. pennsylvanicus*, respectively) are non‐monogamous and do not exhibit biparental rearing of offspring.^41^ In contrast, the prairie vole is well known for its tendencies to form long‐term monogamous pair bonds and to exhibit biparental family structure.^29^, ^37^, ^42^, ^43^ Comparative work between the monogamous and non‐monogamous species has revealed that aspects of neural phenotype account for the different social organizations and mating systems these species demonstrate.^18^, ^44^

Pair bonding in prairie voles is very well documented, and robust behavioral assays have enabled the examination of social and neuroendocrine factors that influence the formation of pair bonds,^42^ which has led to an increasingly well‐developed neurobiological model for mammalian pair bonding (e.g., see References 18, 26, 45, 46). As shown in Box 1, the neural structures that have been closely associated with prairie vole pair bonding include a combination of reward nuclei^47^ and structures that modulate social behavior.^48^, ^49^ This so‐called 'pair bonding neural circuit' represents a subset of a larger network of interconnected forebrain structures (predominately limbic) that are collectively recognized for their general role in modulating social behavior and decision‐making.^50^, ^51^ This 'social decision‐making network' (SDMN) is an ancient network of highly homologous brain areas that has proven valuable in identifying neurobiological mechanisms involved in the regulation of social behavior across vertebrates. Arguably, the decision to form a pair bond falls safely within social decision‐making, and it is reasonable to consider the pair bonding neural circuit as a sub‐unit of the SDMN from both a behavioral and neuroanatomical point of view.

**BOX 1:**
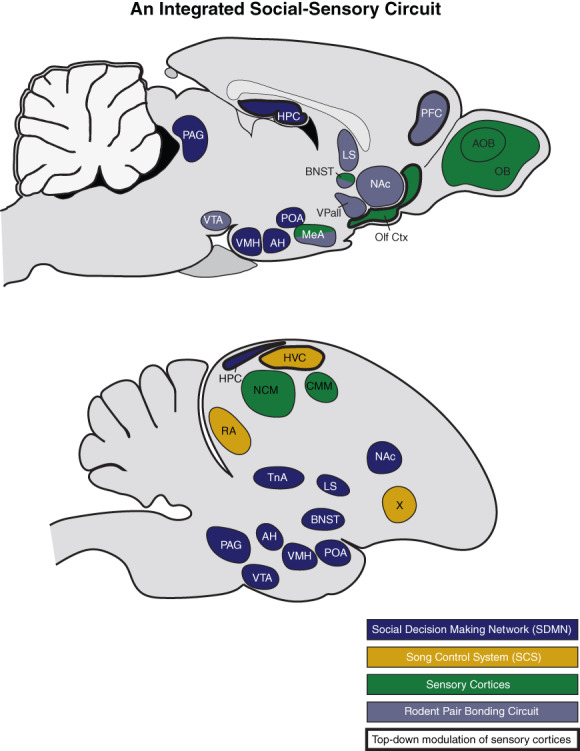
Defining the brain networks that support social communication and behavior has tremendous value for elucidating brain function and supporting broad translational applications for human mental health and well‐being. The foundations and trajectories of prairie vole and zebra finch neurobiological research are quite different, but highly complementary. Together these two well‐studied species offer complementary perspectives on what an integrated social‐sensory communication circuit might look like. We believe that an integrated network including the well‐described network associated with the control of social behavior, known as the social decision‐making network (SDMN), in addition to neural circuitry responsible for chemosensory and auditory processing within the rodent and avian brains provide a platform for envisioning such an integrated social‐sensory communication circuit. The SDMN (denoted with blue) is a large network of interconnected forebrain (primarily limbic) and midbrain structures that are collectively recognized for their general role in modulating social behavior and decision‐making.^51^ The mammalian pair bonding circuit (denoted with gray) largely represents a subset of the SDMN nodes, and is comprised of olfactory processing areas of the brain, reward nuclei,^47^ and structures that modulate social behavior.^48^, ^49^ Additionally, the avian song control system (SCS; denoted with yellow) represents a well‐documented set of specialized brain regions that are central for the production and perception of vocal communication among avian species. The full pair bonding circuit and SCS are described below for context. Key sensory cortices are highlighted in green (related to auditory processing in zebra finches and chemosensation in voles). *Rodent pair bonding circuit*: At its core, the prairie vole pair bonding neural circuit combines reward with facilitated learning of social identity (based on olfactory information) to enhance the social valence of a particular conspecific, resulting in pair bond formation.^60^ According to this model, mating behavior stimulates the ventral tegmental area (VTA) to activate dopaminergic reward systems in the prefrontal cortex (PFC) and the nucleus accumbens (NAc), while simultaneously stimulating the release of the neuromodulatory peptides oxytocin and vasotocin (OT and VT respectively, see)^59^ within the PFC, NAc and ventral pallidum (VPall).^36^ Concurrent with the release of OT and VT, chemosensory information originating from a mate is detected by sensory neurons in the main olfactory and vomeronasal sensory epithelia, initially processed in the olfactory bulb (OB) and accessory olfactory bulb (AOB), then projected to a number of (mostly cortical) structures within the olfactory pathway (olfactory cortex, Olf Ctx) before being integrated in the medial amygdala (MeA).^73^ In response, the MeA and the bed nucleus of the stria terminalis (BNST) simulate release of VT in the lateral septum (LS). The presence of OT in the MeA and VT in the LS facilitate chemosensory learning of the specific chemical signature of each partner's respective mate.^26^, ^60^ This concurrent activation of the reward system with facilitated sensory learning results in a conditioned partner preference (i.e., a pair bond). The prairie vole pair bonding circuit highlights a clear circuit that integrates sensory‐social information (OB/AOB ‐>Olf ‐> MeA). However, as discussed throughout the paper, the extent to which this circuit is involved after initial pair bonding, and/or the extent to which neural circuits shape perception and social bonding (including top‐down modulation of sensory processing) remains largely unexplored. *Avian song control system (SCS)*: The SCS is a series of discrete brain nuclei involved in the production and learning of vocalizations. The SCS has traditionally been studied with respect to male song. This SCS neural circuit includes two primary pathways. The first is the motor pathway comprised of the HVC (proper name), which projects to the robust nucleus of the arcopallium (RA). The RA, in turn, projects to tracheosyringeal portion of the hypoglossal nucleus (nXIIts), which is involved in motor projection in the syrinx. This pathway is required for normal vocal (predominately song) production throughout life. The motor pathway also intersects with the auditory regions of the avian pallium, which is analogous to the mammalian auditory cortex. These auditory regions consist of the caudomedial nidopallium (NCM) and caudomedial mesopallium (CMM) via reciprocal projections through the nucleus interfacialis (NIf). HVC also projects to the second primary pathway in the SCS ‐ the anterior forebrain pathway. In this pathway, HVC projects to Area X (homologous to mammalian basal ganglia and a strong target of midbrain dopamine projections), the medial nucleus of the dorsolateral thalamus (DLM), and the lateral magnocellular nucleus of the anterior neostriatum (LMAN; a frontal cortex‐like nucleus). The two pathways again interact via projections from LMAN to RA.^125^, ^131^, ^219^ In this diagram, we highlight the nuclei from the SCS that have been directly and broadly implicated in acoustic communication (HVC, RA, and Area X). There are numerous candidate pathways that putatively integrate information between the SDMN and SCS – here we highlight VTA‐Area X^162^, ^163^ and mPOA ‐> PAG ‐> HVC.^164^, ^165^, ^169^ For both species, we include the interconnected social behavior circuit and key dopaminergic projections to this circuit (together making the SDMN). These include (1) the hypothalamic structures ‐ ventromedial hypothalamus (VMH), anterior hypothalamus (AH), and the preoptic area (POA), and (2) reward circuitry ‐ periaqueductal gray (PAG), and the medial amygdala (MeA) in mammals or the nucleus taenia (TnA) in birds. Beyond initial pair bonding, the broader SDMN is likely involved in social decision‐making more broadly and may be crucial for regulating pair‐directed behavior and social cognition throughout the lifetime. Further research focused on the functional connectivity between the SDMN and communication circuits promises to reveal a more comprehensive view of the neural mechanisms subserving social behavior. Finally, we highlight key brain regions that function in top‐down neuromodulation of sensory processing (denoted with bold outline). In voles, although the PFC is accounted for in the pair bonding circuit, other regions such as HPC and Olf Ctx are not. These ‘top‐down’ areas that are often not included in efforts to understand pair bonding merit closer attention if we are to achieve a more complete understanding of the neural processing of this important social behavior (e.g., see Reference 220)

Although prairie voles typically form strong and enduring pair bonds, there remains extensive individual variation in the extent and expression of social bonding^52^ – a phenomenon that has received relatively less attention. Notably, prairie voles exhibit alternative mating tactics in nature, in some cases either remaining single or forming pairs and mating outside the pair opportunistically.^29^ The alternative reproductive strategies among vole species and alternative mating tactics observed within species demonstrates that profound variability exists within the social structures of voles. Thus, this system allows deep exploration of the mechanisms underlying reproductive decision‐making and social bonding. Here, we discuss mechanisms of social communication among prairie voles in the context of pair bonding to illustrate the dynamic relationship between sensory systems and social cognition in mammals.

### 2.1 Chemical communication and pair bonding

It is well established that chemical communication plays an essential role in many aspects of social communication and reproduction in rodents.^53^, ^54^ The chemosensory system is crucial for bonding because the expression of a pair bond requires the ability to recognize and remember the specific identity of a mate.^55^ In prairie voles, like most rodents, social recognition depends on chemosensation of an individual's chemical profile. Thus, the chemosensory pathway provides the fundamental information necessary for the neural circuitry of pair bonding.^26^ Notably, prairie voles do not pair bond without an intact olfactory bulb^42^, ^56^ or a functional vomeronasal organ.^57^, ^58^ The presence of oxytocin (OT) in the medial amygdala (MeA) and vasotocin (VT, see)^59^ in the lateral septum (LS) facilitate chemosensory learning of the specific chemical signature of each partner's respective mate.^26^, ^60^ This concurrent activation of reward systems with facilitated sensory learning results in a conditioned partner preference, that is, a pair bond.

The relationship between sensory input and behavior is commonly conceptualized as unidirectional (i.e., sensory input leads to behavioral output). However, this perspective falls far short of accurately describing the dynamic nature by which sensory information influences, and in turn is influenced by, the ongoing social and sensory experiences associated with social bonding. Although being bonded requires social recognition of one's mate,^55^ the very ability to discriminate any particular chemical profile within the extremely complex chemical environment in which an animal lives is dependent upon continual refinement of a dynamic system of signal processing and chemosensory learning.^61^ Furthermore, it is likely that chemosensory communication between pair bonded partners is important beyond initial partner preference formation. Across rodents, chemical signals are known to encode a staggeringly diverse array of behaviorally and ecologically relevant information. Chemical signaling is known to facilitate detection and evaluation of reproductively available potential mates to maximize fecundity,^62^, ^63^ detection and avoidance of sick or parasitized conspecifics,^64^, ^65^ evaluation of age,^66^ quality of diet,^67^ and levels of stress or hunger in nearby conspecifics,^68^, ^69^ as well as detection and avoidance of predators or other dangerous environmental factors.^70^, ^71^, ^72^ Clearly, there is the potential for a tremendous amount of behaviorally relevant information to be encoded in chemosignals beyond sex and individual identity. The importance of chemical communication between pair bonded partners, beyond social recognition and partner preference formation, remains largely unexplored.

### 2.2 Neurobiological regulation of chemosensation during pair bonding

As shown in Box 1, the olfactory bulb (OB) is included in the mammalian pair bonding circuit, and it has long been recognized that information from chemical signals is important for social bonding. Chemosensory information originating from a mate is detected by sensory neurons in the main olfactory and vomeronasal sensory epithelia. This information is initially processed in the olfactory bulb and accessory olfactory bulb, then projected to a number of (mostly cortical) structures within the olfactory pathway before being integrated in the MeA.^73^ Ultimately, the diversity of individual chemical ligands that can be detected by chemosensory systems is based on the genetic repertoire of receptor genes in the genome. The functional repertoire of receptor proteins present in the sensory epithelium is adaptively established by control of gene expression^74^ and experience‐based survival of sensory neurons^75^ resulting in functionally optimized sensory receptor profiles. However, this description does not accurately portray the dynamic nature of chemosensory processing. Decoding raw chemosensory input is an extremely complex task requiring multiple mechanisms working in concert to filter unwanted information and increase signal‐to‐noise ratios to discriminate functionally relevant signals embedded in a complex background.^76^, ^77^, ^78^ To accomplish this task, a wide array of functionally diverse neuromodulatory mechanisms is employed at multiple levels within the chemosensory pathway.

A great many genes are involved in chemosensory processing and producing behavioral responses to chemical stimuli. Thus, variation in genetics and mechanisms governing gene expression have the potential to greatly impact the function of this system. To cite just one illustrative example, consider the oxytocin receptor (OTR), which is encoded by a single functional gene (*otr*). Recall that OT is necessary and sufficient for pair bonding in prairie voles.^26^ Unsurprisingly, the presence and density of the OTR protein has a large impact on vole social behavior. OTR expression within the mesolimbic pathway is required for pair bonding.^18^ Additionally, region‐specific densities of OTR predict social organization between monogamous and non‐monogamous voles species,^44^ and manipulation of OTR density via pharmacological or genetic means alters pair bonding.^79^, ^80^, ^81^, ^82^ In addition to mediating social behavior, OT and OTR play a key role in neuromodulation and early signal processing within the olfactory pathway.^77^ Furthermore, OT‐OTR binding in the rat OB enhances the signal‐to‐noise ratio during olfactory information processing resulting in improved olfactory specificity and discrimination of social signals.^77^ Behaviorally, this results in increased exploration of social chemical signals during interactions with conspecifics and enhanced social recognition.^77^ Thus, the release of OT and the presence and density of OTR accomplish several functions, including asserting critical control over pair bonding, and modulating the OB to enhance sensory information processing and social recognition.

The OB itself contains intrinsic neuromodulatory mechanisms and memory circuitry capable of modifying sensory information based on prior experience. Thus, the OB is able to perform a surprising amount of signal processing prior to sending the information to other areas of the brain.^76^ Mitral cells and periglomerular cells in the OB receive input directly from olfactory sensory neurons and process the signal before relaying information to the piriform cortex and other higher order processing areas.^76^, ^83^, ^84^, ^85^ As sensory input is processed through the OB, inhibitory granule cells act to modulate, dampen, and filter the output from mitral cells to increase the signal to noise ratio. Olfactory learning is accomplished as sensory information guides the development of granule cells, such that only specific mitral cells are inhibited thereby resulting in cleaner signals representing learned odorant profiles.^76^, ^85^ These intrinsic memory circuits are maintained and expanded upon as adult neurogenesis incorporates new granule cells into the OB, which may subsequently be arranged to include additional odorant profiles.^85^

In addition to intrinsic mechanisms, the OB receives a great deal of extrinsic top‐down neuromodulatory input from a diverse range of brain regions involved in memory formation such as the hippocampus (HPC, via the lateral entorhinal cortex), anterior olfactory nucleus, piriform cortex, and prefrontal cortex (PFC).^86^, ^87^ Cortical projections from these regions primarily activate a subset of granule cells within the OB, which act to further modulate and filter sensory information based on learned odorant profiles.^88^

Memory circuits also exist further along the chemosensory pathway including in the MeA, bed nucleus of the stria terminalis (BNST), LS, nucleus accumbens (NAc), and PFC, which further modulate sensory information while simultaneously functioning to produce appropriate behavioral responses to social chemical signals.^26^, ^89^, ^90^ Although the olfactory pathway is affected by multiple neuromodulatory mechanisms, the extent to which these mechanisms shape and are impacted by emerging pair bonds remains unclear.

### 2.3 Behavioral evidence for enduring effects of pair bonding on social recognition

Behavioral evidence provided by prairie voles supports the hypothesis that social bonding produces lasting impacts on chemosensory processing and social cognition. Blocker and Ophir (2015) showed that paired male voles exhibit social recognition of individual females based on chemical profiles, whereas single voles (i.e., un‐paired) do not discriminate between individual females.^91^ Paired voles presumably benefit from enhanced individual recognition of females, as pair bonding inherently requires partners to recognize and remember the individual to which they are bonded. Likewise, single voles might benefit from indiscriminate mating, which does not require social recognition of individual females. These findings suggest that differences in sensory processing among single and paired voles affect social recognition of potential mates, although the specific neural mechanism underlying this shift in social cognition is still unknown.

Given how important chemosensory information is for prairie vole social behavior, the fact that social recognition is fundamental to pair bonding is easily taken for granted such that it is assumed to simply be a static foundational component of a pair bond. However, behavioral studies call into question this assumption, and more readily support the hypothesis that the ability to recognize a conspecific is dynamic, depending on social and environmental context. This is evident when examining social recognition abilities of *single* male voles.^92^ Zheng et al., (2013) demonstrated that the interpretation of social chemical signals by prairie voles depend on the social and environmental context in which they were sensed. In this study, single male voles demonstrated social recognition using a habituation‐dishabituation paradigm to assess discrimination between individual males. Zheng et al. (2013) found that male's social recognition of other males was most pronounced in a familiar environment, and weakest in a novel environment absent of social cues. Consistent with Blocker and Ophir (2015), single males did not discriminate between females, but they did discriminate between males, indicating contextual valence and/or motivation to attend to social cues in a sex‐dependent manner. This result is even more peculiar considering that chemical profiles of females are known to convey sufficient information to allow social recognition and individual discrimination.^91^ Thus, key differences in processing and interpreting chemosensory information not only exist, but they are particularly sensitive to social context. The emphasis on male, but not female recognition among single males suggests the existence of a mechanism that favors individual recognition of potential neighboring competitors while also favoring indiscriminate mate selection. Moreover, such a mechanism can be particularly beneficial for male voles that do not have a pair‐mate.^29^ Although the specific neuromodulatory mechanisms underlying these observations have not yet been elucidated, OT and VT are well known to influence the recognition process.^93^ This is one example of how dynamic chemosensory perception shapes social behavior phenotypes, and these behavioral findings constitute evidence that adaptive sensory modulation impacts reproductive decision‐making in prairie voles.

### 2.4 Enduring effects of pair bonding on chemosensory processing

What neurobiological mechanisms might account for the contextual effect of social bonding on social recognition? Mating, exposure to chemical social signals, and formation of pair bonds are often associated with long‐term mechanisms of neuroplasticity in key structures within the chemosensory processing pathway and in the SDMN. The well‐characterized “Bruce effect”, in which pregnancy is spontaneously aborted after exposure to chemical cues from an unfamiliar male,^94^ provides an intriguing example. Specifically, the Bruce effect is normally avoided by chemosensory imprinting driven by adult neurogenesis in the accessory olfactory bulb (AOB) occurring in response to continual exposure to familiar male chemical cues.^95^ Hence, this illustrates the powerful effect neuroplasticity has on physiology and behavior. Indeed, adult neurogenesis provides a mechanism of neural plasticity allowing adaptive optimization of neural circuitry to meet current social or environmental demands.^96^, ^97^ In prairie voles and another socially monogamous rodent (*Mus spicilegus*), adult neurogenesis is observed in the MeA following formation of a pair bond.^98^, ^99^ Further evidence from prairie voles demonstrates that mating and exposure to social chemical signals stimulates neurogenesis in the subventricular zone (SVZ), dentate gyrus (DG) of the hippocampus, and the rostral migratory stream (RMS), which produce neurons ultimately destined for the OB^100^ and AOB.^83^ In contrast, social isolation reduces neurogenesis and survival of neurons in the MeA, medial preoptic area of the hypothalamus (mPOA), and the DG and ventromedial HPC.^101^ It remains unclear what functions this impressive neuroplasticity may serve beyond initial pair bonding.

Neuroplasticity within these circuits may also exist at the level of gene regulatory networks, which reflect and result in changes in neuronal functioning. Pair bonding is associated with robust changes to gene expression in the NAc of prairie voles. Notably, in addition to its critical role in pair bond formation, the NAc is highly interconnected with the olfactory tubercle, which receives input directly from the OB,^102^ providing yet another opportunity for sensory integration with the processing of social behavior. Patterns of gene expression in the NAc are significantly altered during early bond formation occurring within the first 24 h of cohabitation and continue to change during the first several weeks following formation of a bond.^79^ The subset of differentially expressed genes identified during early bond formation only partially overlap with those identified three weeks after formation of a bond, suggesting that separate transcriptional mechanisms have roles within early pair bond formation and later during bond maintenance. The transcriptional profiles associated with pair bond maintenance are stable from two to at least six weeks after bond formation, but these profiles erode within four weeks if partners are separated, suggesting that transcriptional mechanisms underlying pair bond maintenance persist as long as the partners remain paired.^103^ Gene expression affecting pair bond formation may also be altered on a more permanent basis via epigenetic modifications within regulatory regions of the genome. To illustrate this, we again refer to the earlier example involving OT and OTR interactions. Epigenetic modifications within the prairie vole OTR gene promoter region within cells of the NAc increase OTR density, and thereby increase sensitivity to OT and facilitate pair bond formation in both males and females.^79^, ^80^ Although pair bonding can have both profound transient and enduring impacts on pair bonding circuitry, it remains unknown whether and to what extent this occurs within the OB and other chemosensory processing circuitry.

#### 2.4.1 Next steps

The importance of chemosensation in rodents has long been known, and the OB is included in the mammalian pair bonding circuit. Nonetheless, there are striking gaps in our basic knowledge about the role of chemosensory communication beyond social recognition for pair bonded individuals. Moreover, as highlighted above, the transduction and interpretation of chemosensory information is not as straightforward as it may seem. Sensory information is modified by multiple functionally disparate neuromodulatory mechanisms acting in concert to adaptively optimize the social interpretations of chemical signals in intricate ways that we are just beginning to understand. Each of these diverse mechanisms are individually capable of modulating sensory processing. In a living animal, however, most of these mechanisms simultaneously and continuously modify neural activity.^84^ This dynamic nature of chemosensory processing means that the neural representation of a particular chemical profile is highly plastic and adaptive, and it is dependent on multiple factors such as prior experience and learning, environmental and social context, and current physiological state.^88^ This system of feedback adaptively and effectively allows discrimination of minute, but often critical, differences among chemical signals driving social decision‐making. The extent to which pair bonding shapes sensory processing within the OB and chemosensory circuity remains unknown.

## 3 ZEBRA FINCHES

Zebra finches are small songbirds native to the arid and semi‐arid regions of Australia.^104^ The natural ecology and ethology of the zebra finch makes them an excellent system for studying sociality and communication. Zebra finches are highly gregarious, form life‐long sexually monogamous pair bonds, divide parental care equally between partners, and breed opportunistically – one of many adaptations to evolving within extreme and unpredictable climate and environmental conditions.^104^, ^105^, ^106^, ^107^, ^108^ Zebra finches are also one of the most well‐studied avian species in the lab or field.^109^, ^110^ Zebra finches are a predominant study system for a range of biological disciplines including genetics/genomics, physiology, behavior, ecology and neuroscience.^109^, ^110^ Indeed, the second avian genome sequenced was the zebra finch.^111^

Monogamy is overwhelmingly the most common mating system in birds, exhibited by ~90% of species.^112^, ^113^, ^114^, ^115^ Yet even relative to other small songbirds, zebra finches form remarkably strong pair bonds. Zebra finch partners actively maintain their bonds throughout life, even during long non‐breeding periods, and extra‐pair courtship is very rare.^104^, ^106^, ^116^ Zebra finches are one of the most well studied avian species with respect to monogamy and pair bonding.^106^, ^110^, ^116^, ^117^, ^118^, ^119^, ^120^ Like prairie voles, putative mechanisms subserving bonding in zebra finches involve nonapeptide and dopaminergic signaling within brain regions in the SDMN, and research with zebra finches has contributed to our understanding of conserved neuroendocrine mechanisms underlying pair bonding across vertebrates (Box 1).^36^, ^119^, ^121^, ^122^, ^123^

The most profound contribution of zebra finches to neuroscience research has been in establishing a basic understanding of the neurobiology and neurogenomic mechanisms underlying communication and vocal learning.^109^, ^110^, ^124^, ^125^, ^126^, ^127^, ^128^, ^129^, ^130^, ^131^ Juvenile male zebra finches undergo sensorimotor vocal learning, during which they learn to produce mature adult vocalizations (male song and distance calls) via exposure to and interaction with adults.^125^, ^131^, ^132^, ^133^ Supporting song learning and behavior, zebra finches have well described neural circuitry referred to as the ‘song control system’ (SCS). Briefly, the SCS contains two primary pathways: (1) a motor pathway essential for vocal production throughout life and (2) the anterior forebrain pathway that appears more specialized for learning. Both pathways begin with the HVC (used here as the proper name), which directly receives input from the avian auditory cortex (Box 1). Although the SCS has been primarily studied for its role in regulating male song learning and production, there is a growing understanding that it is important for communication more broadly.^134^, ^135^, ^136^, ^137^ For zebra finches, acoustic communication is an essential modality for pair bond formation and maintenance. Combined, zebra finches have the potential to be a powerful model system for furthering our understanding of how communication and pair bonding are integrated.

### 3.1 Acoustic communication and pair bonding

For zebra finches, acoustic communication is essential for the process of pairing at every stage. The reliance of zebra finches on acoustic communication for pairing was originally demonstrated decades ago.^120^ Unlike in most rodents, physical contact and copulations are not essential for the formation of pair bonds in zebra finches. Strikingly, some zebra finches will form selective pair bonds without any physical or visual contact if they are able to interact acoustically.^120^ Furthermore, established male‐female pairs appear to easily maintain strong bonds if they remain in acoustic contact with their partner, even within the presence of novel opposite sex individuals.^120^ Although zebra finches clearly rely on acoustic communication to mediate pair bond formation and maintenance, the behavioral mechanisms (e.g., what types of vocal behaviors) involved remain unclear.

The large zebra finch vocal repertoire includes both male song, and male and female calls.^104^, ^118^ Substantial research exists on the importance of male song for mate attraction and female choice in zebra finches and other songbirds.^104^, ^138^, ^139^, ^140^, ^141^ There is considerably less research on the large repertoire and use of male and female calls. Zebra finches produce ~10 categories of calls across a wide range of contexts.^118^, ^142^ Importantly, zebra finches have a specialized ability to discriminate subtle variation in the acoustic fine structure within their vocal signals.^6^, ^7^, ^143^ Similar to chemosensory information in rodents, zebra finch song and calls both carry behaviorally relevant information, including individual identity and internal state (e.g., breeding readiness and stress).^144^, ^145^, ^146^ The intricacies of how such behaviorally‐relevant information is embedded within the auditory signal are beyond human perception.^6^, ^143^, ^147^, ^148^ This suite of vocal behavior and perceptual abilities is critical at one point or another throughout pair bonding.

Zebra finch pair bonding can take up to 2 weeks, however, it is typically assumed to occur much more quickly (on the order of hours to days).^104^ Pair bonding in zebra finches, as in other songbirds, is usually thought to include three stages: (1) a brief courtship phase, (2) a short pair formation phase, and (3) an indefinite pair maintenance phase.^149^, ^150^, ^151^, ^152^ Unlike in rodents, the courtship phase is extremely well studied. Yet, what distinguishes these stages and how long they last remains unclear. For example, pair maintenance encompasses anything that occurs after the establishment of a pair bond. Acknowledging these distinct periods provides a useful basis for discussing how acoustic communication supports and is shaped by pair bonding.

When establishing a pair bond, male song is essential for courtship, and females select mates based heavily on these songs.^104^, ^138^, ^139^, ^140^ Males that are unable to sing are unlikely to successfully attract a mate.^153^ However, female zebra finches do not simply make mate choice decisions based on static assessements of a male signal. Successful courtship involves dynamic vocal interactions, where the female calls extensively during male song and the male responds to this female feedback.^154^ During these dynamic courtship interactions, the characteristically stereotyped and stable male song becomes extremely variable as the male increasingly aligns to the female's vocal behavior.^154^ Such dynamic interactions highlight the intrinsic interplay between song perception and social decision‐making.

After initial pairing, dynamic intra‐pair calling appears to be the most important form of acoustic communication.^154^, ^155^, ^156^ Male song likely does support coordination among pair bonded partners.^157^ Despite this, males that cannot sing are successful at maintaining pair bonds.^158^ Therefore, song is clearly not essential, and the function of song after pairing remains unclear. In contrast, zebra finches rely heavily on intra‐pair calling throughout the pair maintenance phase. Patterns within intra‐pair calling change over the first week of paring,^150^, ^155^, ^156^, ^159^ and partners appear to align behavior to each other more closely. Partners also rely on this form of acoustic communication to coordinate several activities, including biparental care.^118^, ^160^, ^161^ From these findings, we can draw the conclusions that (1) communication and social bonding are clearly merged processes at a behavioral level, (2) pair bond formation and maintenance depend on acoustic communication, and (3) the process of pairing shapes patterns of communication between partners.

### 3.2 Neurobiological mechanisms underlying interactive vocal dynamics

There are rich lines of research on the behavioral integration of acoustic communication and pair bonding in zebra finches. However, unlike in the prairie vole system, no ‘pair bonding neural circuit’ has been described that integrates the SDMN with acoustic communication circuits (SCS and auditory cortex). Despite this lack of a formal integration, broader research on acoustic communication in songbirds highlights potential neurobiological mechanisms that would support such an integration between vocal behavior and social bonding (e.g., social motivation and reward more broadly).

Despite the lack of a described pair bonding circuit, there are numerous candidate pathways that putatively integrate information between the SDMN and SCS. Area X and the ventral tegmental area (VTA), for example, support self‐evaluation during song learning.^162^ In zebra finches, the same neurons projecting from the VTA to Area X also integrate female vocal feedback during courtship in adults.^163^ This Area X‐VTA circuit might support integration of social feedback into communication more broadly. Furthermore, whereas the SCS regulates singing, the *motivation* to sing is primarily controlled by the SDMN. Thus, the medial preoptic area (mPOA) is another possible point of integration between these two systems, because the mPOA regulates motivation to sing.^164^, ^165^, ^166^, ^167^, ^168^ These point of confluence raises the question of how motivational information is relayed between the SDMN and the SCS. One intriguing possibility is that dopaminergic projections from periaqueductal gray (PAG – specifically A11) to HVC function to relay motivational information to the SCS.^169^, ^170^ Continued research regarding the role of these networks in regulating acoustic communication between partners promises to reveal a more comprehensive view of the neural mechanisms subserving social behavior.

Thus far, our best understanding of the neurobiological mechanisms underlying intra‐pair vocal dynamics comes from research in the SCS. There is growing evidence indicating that the SCS has a more general role in supporting social cognition and behavior, beyond simply regulating male song.^134^, ^135^, ^136^, ^137^ It is important to point out that a significant barrier in our progress toward understanding the role of SCS in communication has been the incorrect historical assumption that females do not sing,^171^, ^172^, ^173^ and that song is the most important vocal behavior.^141^, ^174^, ^175^ Nonetheless, we can gain insight into the role of HVC for intra‐pair vocal dynamics from elegant work in the duetting plain‐tailed wrens (*Pheugopedius euophrys*). Electrophysiology recordings on duetting pairs of plain‐tailed wrens, have revealed that HVC regulates song production similarly in both males and females, and that neuronal firing is tightly synchronized and alternated between partners.^176^, ^177^, ^178^ Because HVC integrates auditory information, recordings of HVC neurons can be made in response to auditory input alone. Typically, HVC neurons respond most robustly to recordings of the bird's own song. However, for the plain tailed wrens, HVC neurons of both males and females respond more robustly to hearing the entire duet than to simply their individual contribution.^177^ In this way, the entire duet, and its neural representation in HVC, reflects the extent to which social behaviors can be distributed between partners.

In zebra finches, the SCS has also been implicated in the regulation of intra‐pair calling dynamics.^135^, ^179^, ^180^ This is particularly striking because the SCS is predominately studied with respect to song – even when considering that the SCS is important for female acoustic communication.^137^, ^181^ Even though female zebra finches do not sing, and their SCS nuclei volumes are smaller than in males, the female SCS is functional and has connectivity comparable to that of the male SCS.^182^ In fact, the production and timing of both female and male calls appear to be regulated by SCS (particularly by the HVC, the robust nucleus of the arcopallium (RA), and their projections), and females are more flexible ‘communicators’ than males, demonstrating a greater ability to modulate the timing of their calls.^135^, ^179^, ^180^ The extent to which this pathway may be specialized or shaped by pair bonding remains unknown.

### 3.3 Enduring effects of pair bonding on auditory perception

Pair bonding has enduring impacts on brain and behavior. Again, a striking example of such an enduring impact is social recognition for a mate. Auditory recognition of a mate is essential for zebra finch pairs, just as prairie vole pairs use olfactory cues to identify their mates. Social experience and signal salience are hypothesized to be encoded within the auditory region the caudomedial nidopallium (NCM).^183^ In females, the neuronal spiking activity in NCM is higher in response to hearing a familiar call^183^, ^184^ and V1aR density is higher in NCM after pairing.^185^ Furthermore, temporary inactivation of the female NCM decreases song and mate choice discrimination and modulates patterns of affiliation between partners.^186^ Finally, in both male and female NCM, patterns of NCM neuronal activity are modulated in behaviorally relevant ways based on call type, the identity of the sender, and the breeding context.^184^ Such plasticity within the NCM demonstrates how interactions between social and sensory experiences alter brain anatomy and function.

Not only do zebra finches learn to recognize their partner's ‘voice’, but they also develop a preference for their partner's song and calls. In an operant behavioral paradigm, it is clear that females find both their mate's call and song are both rewarding.^187^, ^188^ Neuroplasticity within NCM is also important for governing these preferences. More specifically, NCM reflects the incentive salience of signals and is a putative region responsible for integrating sensory information, motivation, and reward.^189^ Specifically, dopamine signaling via the D1‐receptor within NCM underlies the formation of female preferences for male song.^190^ Whether similar mechanisms underlie male preferences for their partner's vocalizations remains unknown.

Together, these findings highlight the important role of neuromodulators (nonapeptides and dopamine) as key signaling mechanisms underlying the integration of social experience and perception. However, there are numerous other neuromodulatory mechanisms that could influence perception in zebra finches. Estradiol (E2) signaling is another putative neuromodulator that is likely responsible for encoding social experience throughout the auditory system. E2 signaling within NCM has been shown to be important for regulating associative learning and incentive saliency.^191^, ^192^, ^193^, ^194^ Intriguingly, E2 signaling also appears to modulate the saliency of acoustic signals in the auditory cortex and within the inner ear itself.^193^, ^195^, ^196^ It remains unclear whether (and if so, to what extent) E2 signaling throughout the auditory pathway is shaped by pair bonding and how it may regulate pair‐directed behavior.

### 3.4 Conclusions and next steps

Zebra finches are extremely well studied for both pair bonding and acoustic communication, and it has long been known that acoustic communication is essential for pair bonding. Reflecting this knowledge, a tremendous amount of research describes acoustic communication between pair‐bonded partners, which behaviorally highlights the integration of communication and pair bonding processes. However, studies examining the mechanisms underlying the integration of perception, communication, and social bonding have not been extended much beyond behavior to physiological or neurobiological integration. Based on what is known about the ethology of zebra finches, we speculate that individual differences in perception impact pairing, and that social bonding has enduring impacts on patterns of perception and communication. Substantial effort has provided a strong understanding of the neurobiological substrates of vocal communication in birds, particularly zebra finches. This knowledge should be integrated with social bonding research because it promises to enrich the ways we understand the function of the social brain. Lack of this integration has left profound gaps in our knowledge, and key questions remain unanswered – such as (1) how does pair bonding impact the SCS and support communication between partners, (2) what neurobiological mechanisms support dynamic non‐song vocal exchanges, and (3) how is information from the SDMN integrated with ‘communication circuits’.

## 4 DEVELOPING AN INTEGRATED SOCIO‐SENSORY FRAMEWORK

**FIGURE 1:**
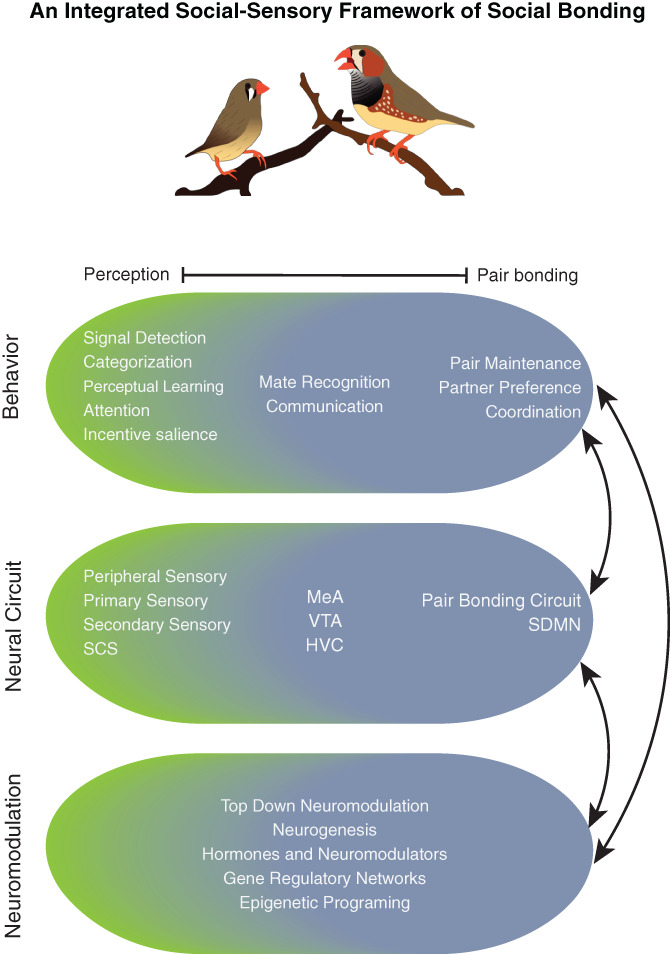
There are entry points across levels of analysis (behavioral, neural circuit/ neuroanatomical, and neuromodulatory) to apply an integrated approach to studying pair bonding. In this schematic, we highlight variables traditionally studied as metrics for sensory/perception (shaded green) and pair bonding (shaded gray), with clear examples of integrated metrics centered in the middle. Examples of integrated metrics include behavioral processes such as mate recognition and intra‐pair patterns of communication, with key brain regions that regulate these processes such as the medial amygdala and HVC. We propose designing experiments that incorporate or specifically control for metrics traditionally associated with sensory/perception and pair bonding. This integration can be done either within or across levels of analysis. Studies focusing on how social experience modulates perception, sensory processing, and communication promise to be particularly powerful for clarifying how social bonding produces lasting impacts on brain and behavior. SCS, song control system; SDMN, social decision‐making network

Communication, perception, and social bonding are interrelated processes. At some level, this interrelatedness may be intuitively obvious. However, the predominant approaches to studying perception and social bonding have been siloed and are typically addressed separately across basic and applied research (or between clinical research and clinical practice in humans). We argue that our understanding of communication, perception, and social bonding will benefit from applying an integrated social‐sensory framework. In this review, we leverage what is known about the zebra finch and prairie vole model systems to highlight how these processes are interrelated at a basic level, and we explore how we stand to benefit from an integrated framework. Pair bonding in particular serves as an exemplar social behavior that also holds special value to human health and wellbeing.^18^, ^46^, ^197^ As discussed above, sensory processing and perception are clearly essential for, and reflect the processes of, pair bonding for both species. For prairie voles, chemosensory signals are integral to pair bond formation and social recognition is necessary for pair bond maintenance. Auditory signals are likewise important to zebra finches, which remarkably can form and maintain pair bonds using acoustic communication alone. Furthermore, there are strong connections between social behavior networks and communication circuits/sensory cortices in both the prairie vole and zebra finch brain. Indeed, the OB has long been identified as a key node in the prairie vole pair bonding circuit. In zebra finches, interactive vocal communication between partners relies on the specialized SCS. By comparing research approaches across these two study systems, we can highlight numerous entry points for integrating social and sensory processes across these levels (Figure 1).

One of the most straightforward ways to apply a social‐sensory integrated approach is to design experiments that combine behavioral manipulations, metrics and/or dependent variables from perception together with those relevant to social bonding (Figure 1). In this way, the effect of pair bonding status on communication, perception, and sensory processing can be accounted for, described, and quantified. For parameters of perception, there are numerous dimensions beyond social recognition, such as assessment of detection and discrimation of signals, categorization, perceptual learning, attention/incentive salience, and working memory. Furthermore, the effects of pair bonding on neuromodulation of sensory cortices and circuits themselves can be quantified. As highlighted above for both prairie voles and zebra finches, neuromodulation exists at every level of sensory processing – including peripheral sensory organs, and primary and secondary sensory cortices. Here, we highlight examples demonstrating that this is happening, but the extent and consequence of social experience‐driven neuromodulation of social signals within the context of social bonding remain largely unknown.

Another way to apply a social‐sensory integrated approach is to focus on the interconnectivity of neurobiological networks, including the SDMN, the pair bonding circuit, and communication neural circuits (Box 1, Figure 1). Such research could focus on functional and direct connections between sensory cortices, communication circuits and social behavior circuits. This approach has dramatically shifted our understanding of behavioral and neurobiological mechanisms of communication when it has been used.^163^, ^198^, ^199^ Applying this approach to pair bonding would involve focusing on neural circuitry beyond brain regions associated with social behavior or the pair bonding circuit. Even in prairie vole research where the OB has long been considered an integral part of the pair bonding neural circuit, it is rarely studied beyond its essential role in social recognition. Just as the SCS is important in regulation of zebra finch intra‐pair communicative dynamics, it is likely that chemosensory processing is playing a crucial role in more dynamic aspects of intra‐pair behavior among prairie voles.

As we consider an integrated social‐sensory framework, it is important to highlight that perception is not a simple pathway consisting of sensory inputs and behavioral outputs. In fact, behaviors are not simply social outputs of perception. Animals can interactively alter their behavior to change their exposure to social signals. Increases to sniffing rates, which improve odor detection and discrimination,^200^ provides one such exmaple for olfaction. Behavioral adjustments that modify perception often occur across sensory modalities. For example, hearing and vision are behaivorally adjusted by altering the orientation of the pinna act to coordinate sound localization,^201^ or by orienting the head or body toward a visual stimulus,^202^ respectively. In other words, dynamic behavioral adjustments functionally alter the salience of a signal. Furthermore, signal processing is not a simple unidirectional pathway where the most salient elements of the signal are perceived, followed by higher‐order cognitive processing, and terminating with a behavioral output. In fact, there exists extensive top‐down neuromodulation that influences perception across peripheral sensory organs and the primary and secondary sensory corticies (e.g., auditory;^203^ visual;^75^, ^204^ and olfactory).^87^ Finally, social signals are multimodal during natural social interactions,^205^, ^206^, ^207^, ^208^, ^209^, ^210^, ^211^ and sensory cortices are processing stimuli across modalities.^212^, ^213^, ^214^ A clear example in human communication is how the auditory perception of human speech can be profoundly impacted by visual perception of lip movements.^214^ This highlights a type of social‐sensory functional integration in and of itself.

Taken together, it is apparent that communicative behaviors are essential for mate choice, courtship, and bond maintenance, and that perceptual processes are shaped by social experience suggesting bi‐directional reciprocal interactions between social behavior and sensory signal processing across species. We have discussed evidence that justifies an explicit integration of social bonding and perception to broaden our concept of the relationships between them. Indeed, an integrated social‐sensory framework will be particularly valuable for addressing several important questions, including (1) how is social bonding shaped by individual perceptual differences?, and (2) how is perception influenced by social experience – particularly social interactions and emerging relationships? Beyond social bonding, there is growing evidence that the effects of social experience on brain and behavior are inextricably linked to the neuromodulation of sensory signals,^199^, ^215^, ^216^, ^217^, ^218^ making this approach broadly applicable across species, contexts, and sensory modalities. Such a unified framework that concomitantnly integrates social and sensory processing will allow us to account for varying levels of dynamic and multimodal complexity in an attempt to more deeply understand the nature of sociality.

## CONFLICT OF INTEREST

The authors declare no potential conflict of interest.

## Data Availability

Data sharing is not applicable to this article as no new data were created or analyzed in this study.
